# Osteogenic impact of pro-apoptotic caspase inhibitors in MC3T3-E1 cells

**DOI:** 10.1038/s41598-020-64294-9

**Published:** 2020-05-04

**Authors:** Adéla Kratochvílová, Barbora Veselá, Vojtěch Ledvina, Eva Švandová, Karel Klepárník, Kateřina Dadáková, Petr Beneš, Eva Matalová

**Affiliations:** 1 Institute of Animal Physiology and Genetics, Academy of Sciences, Brno, Czech Republic; 2 Faculty of Science, Masaryk University, Brno, Czech Republic; 3 Institute of Analytical Chemistry of the Czech Academy of Sciences, Brno, Czech Republic; 4 International Clinical Research Center, St. Anne’s University Hospital, Brno, Czech Republic; 5 Department of Physiology, University of Veterinary and Pharmaceutical Sciences, Brno, Czech Republic

**Keywords:** Cell biology, Proteases, Bone development, Bone

## Abstract

Caspases are proteases traditionally associated with inflammation and cell death. Recently, they have also been shown to modulate cell proliferation and differentiation. The aim of the current research was to search for osteogenic molecules affected by caspase inhibition and to specify the individual caspases critical for these effects with a focus on proapoptotic caspases: caspase-2, -3, -6, -7, -8 and -9. Along with osteocalcin (*Ocn*), general caspase inhibition significantly decreased the expression of the *Phex *gene in differentiated MC3T3-E1 cells. The inhibition of individual caspases indicated that caspase-8 is a major contributor to the modification of *Ocn *and *Phex* expression. Caspase-2 and-6 had effects on *Ocn* and caspase-6 had an effect on *Phex*. These data confirm and expand the current knowledge about the nonapoptotic roles of caspases and the effect of their pharmacological inhibition on the osteogenic potential of osteoblastic cells.

## Introduction

Caspases are proteases that are currently associated with inflammation and cell death. Their use has broad implications for pathological conditions, such as cancer and degenerative disorders. Caspase inhibitors have been tested in several therapeutic approaches^1^. Additionally, a much broader spectrum of caspase functions has been demonstrated^2^, particularly proapoptotic caspases, including apical caspases-8 and-9, the executive trio of caspase-3,-6 and-7 and the still enigmatic caspase-2.

New functions of caspases have also been reported in osteogenesis^3,4^. Bmp4-induced differentiation of osteoblastic MC3T3-E1 cells leads to the activation of caspase-2,-3 and-8 without increasing the apoptosis rate^5^. Pharmaceutical inhibition of caspases reduced Alp activity in MC3T3-E1 cells and the expression levels of osteocalcin, a molecule typically found in osteoblasts^4,5^. Notably, osteocalcin is also used in medical diagnoses as a biochemical marker of bone formation and metabolic risk^6^. Pharmacological caspase inhibitors are considered potential tools in several therapies^1^.

Previous works have focused on the alteration of gene expression during MC3T3-E1 cell differentiation^7,8^, which consists of several phases, including proliferation, differentiation and matrix deposition, accompanied by the production of specific osteogenic factors^9^. Since these phases were first characterized^10^, hundreds of reports have been based on experiments performed using MC3T3-E1 cells. Given their intramembranous origin, MC3T3-E1 cells are recognized as suitable *in vitro* models for direct osteoblastic differentiation and pleiotropic studies^11^.

Based on published results and our preliminary data, we hypothesized that proapoptotic caspases impact gene expression in differentiated MC3T3-E1 cells. The following investigation addresses the effects of caspase blockade on gene expression in differentiated cells. For the first time, a cell bioluminescence-based approach was used to record the activation of individual proapoptotic caspases during MC3T3-E1 cell cultivation. Therefore, the osteogenic profile of MC3T3-E1 cells was evaluated after pharmacological caspase inhibition, and individual proapoptotic caspases crucial for the observed effect were specified.

## Results

### Osteogenic expression varies depending on the culture conditions

**Figure 1:**
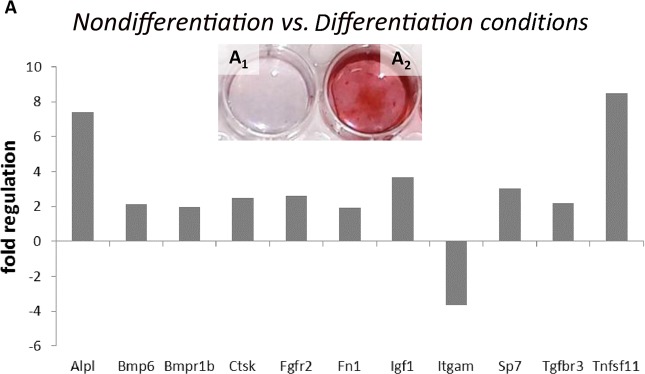
PCR Array analysis of osteogenesis-related gene expression in the MC3T3-E1 cells cultured in nondifferentiation conditions compared to that in cells in differentiation conditions for 21 days (**A**), *p* ≤ 0.05. Alizarin red staining of the cultured cells under the nondifferentiation condition (A_1_) was compared to that of the cells under the differentiation condition(A_2_).

First, we analyzed the osteogenic potential of differentiated MC3T3-E1 cells, which were used as models in our study. Specifically, the expression of a panel of osteogenic genes was compared in cells cultured simultaneously under differentiation and nondifferentiation conditions. The cells were cultured in parallel for 21 days, which was considered the point of complete differentiation^9^. After 21 days, alizarin red staining confirmed the abundant mineralization of the cell matrix cultured in the presence of AA/βGP, an effect not observed in the absence of AA/βGP (Fig. 1A_1_). Out of 84 genes, 11 genes were significantly upregulated or downregulated in the differentiation medium (Fig. 1D), compared to those in the cells cultured in AA/βGP-free medium, and 42 genes were expressed at high levels (Table 1), which did not change, in both groups. Increased expression was detected for*Alpl* (7.4), *Bmp6* (2.15), *Bmpr1b* (1.95), *Ctsk* (2.47), *Fgfr2* (2.6), *Fn1* (1.9), *Igf1* (3.7), *Sp7* (3.02), *Tgfbr3* (2.2) and *Tnfsf11* (8.49),but decreased expression was observedfor *Itgam* (−3.64).

**Table 1:** Osteogenic genes expressed at levels of housekeeping genes (Ct = 15–24) under nondifferentiation and differentiation conditions.

Acvr1	Nfkb1
Anxa5	Pdgfa
Bgn	Runx2
Bmp1	Serpinh1
Bmpr1a	Smad1
Bmpr2	Smad2
Cdh11	Smad3
Col1a1	Smad4
Col1a2	Smad5
Col2a1	Sox9
Col3a1	Spp1
Col4a1	Tgfb1
Col5a1	Tgfb2
Csf1	Tgfb3
Fgfr1	Tgfbr1
Flt1	Tgfbr2
Igf1r	Twist1
Itga3	Vcam1
Itgav	Vdr
Itgb1	Vegfa
Mmp2	Vegfb

There was no change of expression in both groups. Expression was detected by PCR Arrays.

### Apoptotic caspases are activated during differentiation

**Figure 2:**
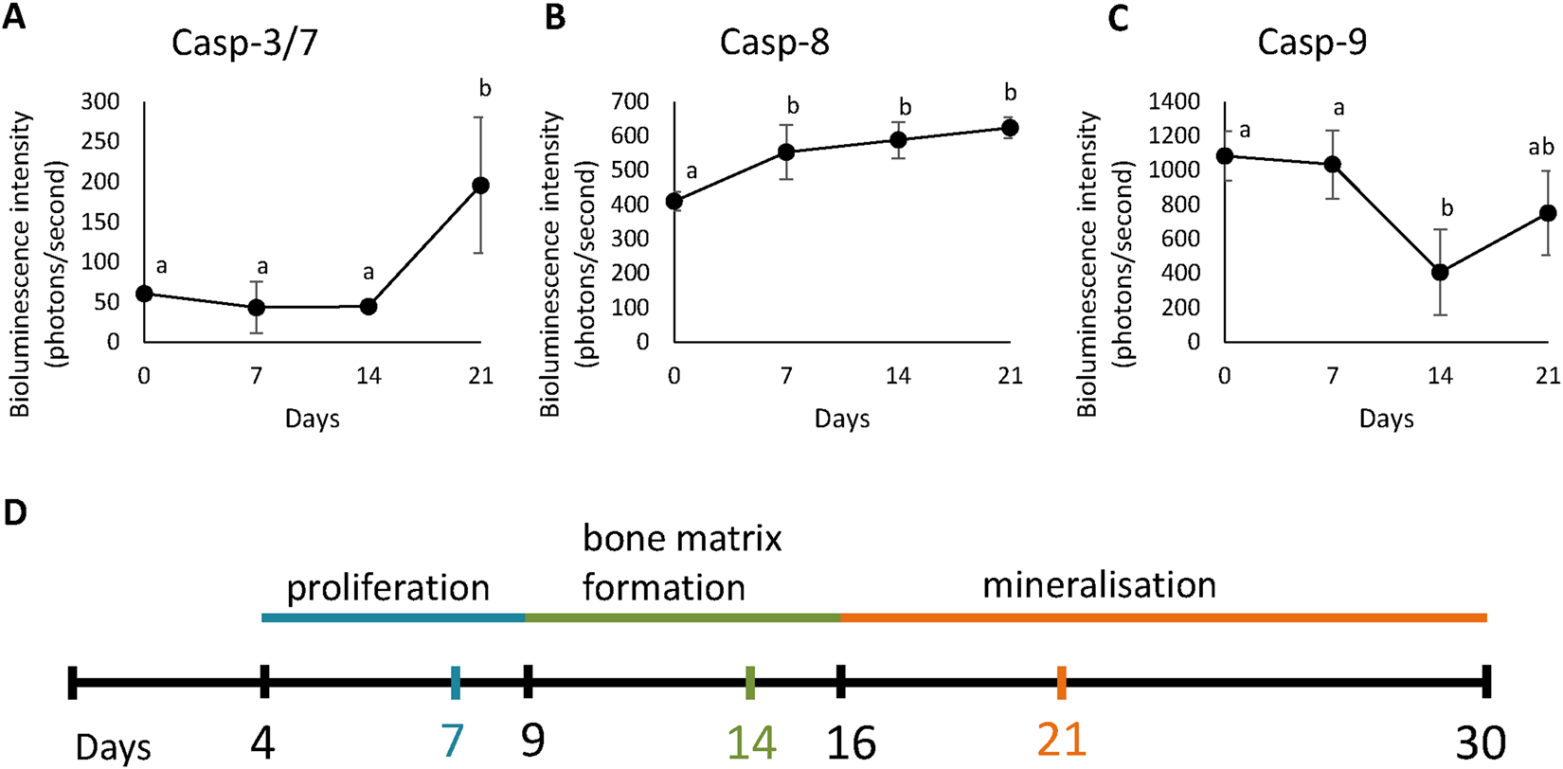
Bioluminescence measures of caspase-3/7,-8 and-9 (**A,B,C**) activity levels in MC3T3-E1 cells cultured under differentiation conditions shown as the means±standard deviations of three replicates. Significant differences are indicated by different letters and related data are listed in Supplement 1. Time scale of the MC3T3-E1 cell differentiation process (by Choi *et al*., 1996^9^) with marks for bioluminescence measurement (**D**).

The activity levels of all investigated caspases were significantly different at the monitored time points (P < 0.05 for all caspases, see Supplement 1). Furthermore, each caspase displayed changes in their activity level at different stages of MC3T3-E1 cell differentiation. At the proliferation stage (between days 0 and 7), caspase-8 activity (Fig. 2B) significantly increased (caspase-8 activity was significantly lower at time point 0 than at all later time points). However, the activity of caspase-9 (Fig. 2C) decreased in the following period (caspase-9 activity was significantly lower after 14 days of the experiment than it was at time points 0 and 7). Caspase-3/7 activity significantly (Fig. 2A) increased at the mineralization stage, between days 14 and 21 (Casp 3/7 activity was significantly higher after 21 days of the experiment than it was at all other time points).

The orientation scale of MC3T3-E1 cell differentiation according to Choi^9^ is shown in Fig. 2D.

### Caspase inhibition changes osteogenic gene expression

**Figure 3:**
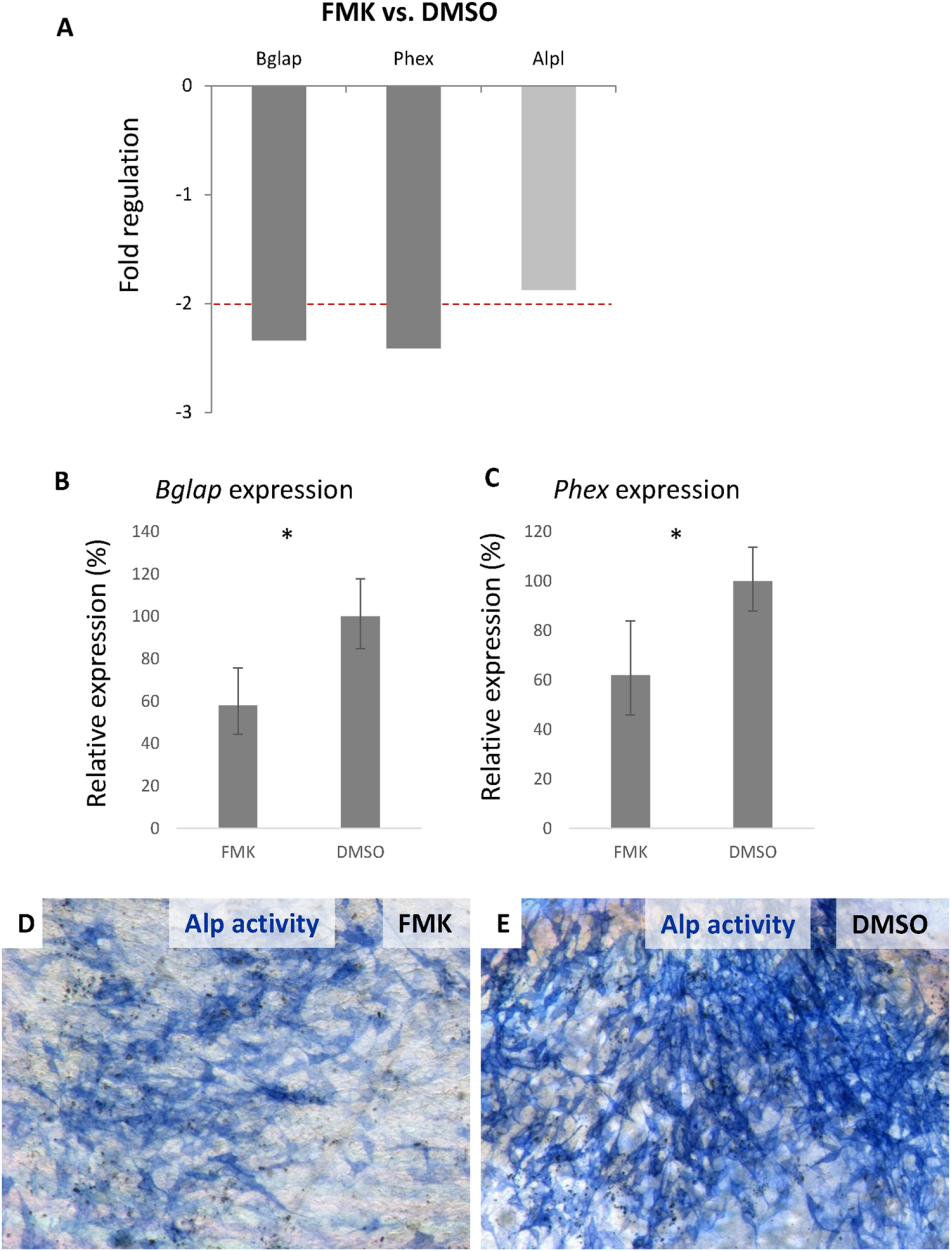
PCR Arrays evaluation of the changes in osteogenesis-related gene expression after six days of caspase inhibition (FMK) compared to that of the control (DMSO), *p* ≤ 0.05, in differentiated MC3T3-E1 cells (**A**). The red line indicates fold changes −2. Expression of *Bglap* (**B**) and *Phex* (**C**) in the differentiated MC3T3-E1 cells was determined by real-time PCR after 6 days of caspase inhibition by the FMK inhibitor. Expression levels were compared to the expression in the control cells.The results are presented as a % indicating the mean ± standard deviation of three replicates (expression in the control cells was set as 100%). * indicates *p* ≤ 0.05. Staining of the alkaline phosphatase activity in FMK-treated (**D**) and control (**E**) cells. Positive cells are blue.

**Figure 4:**
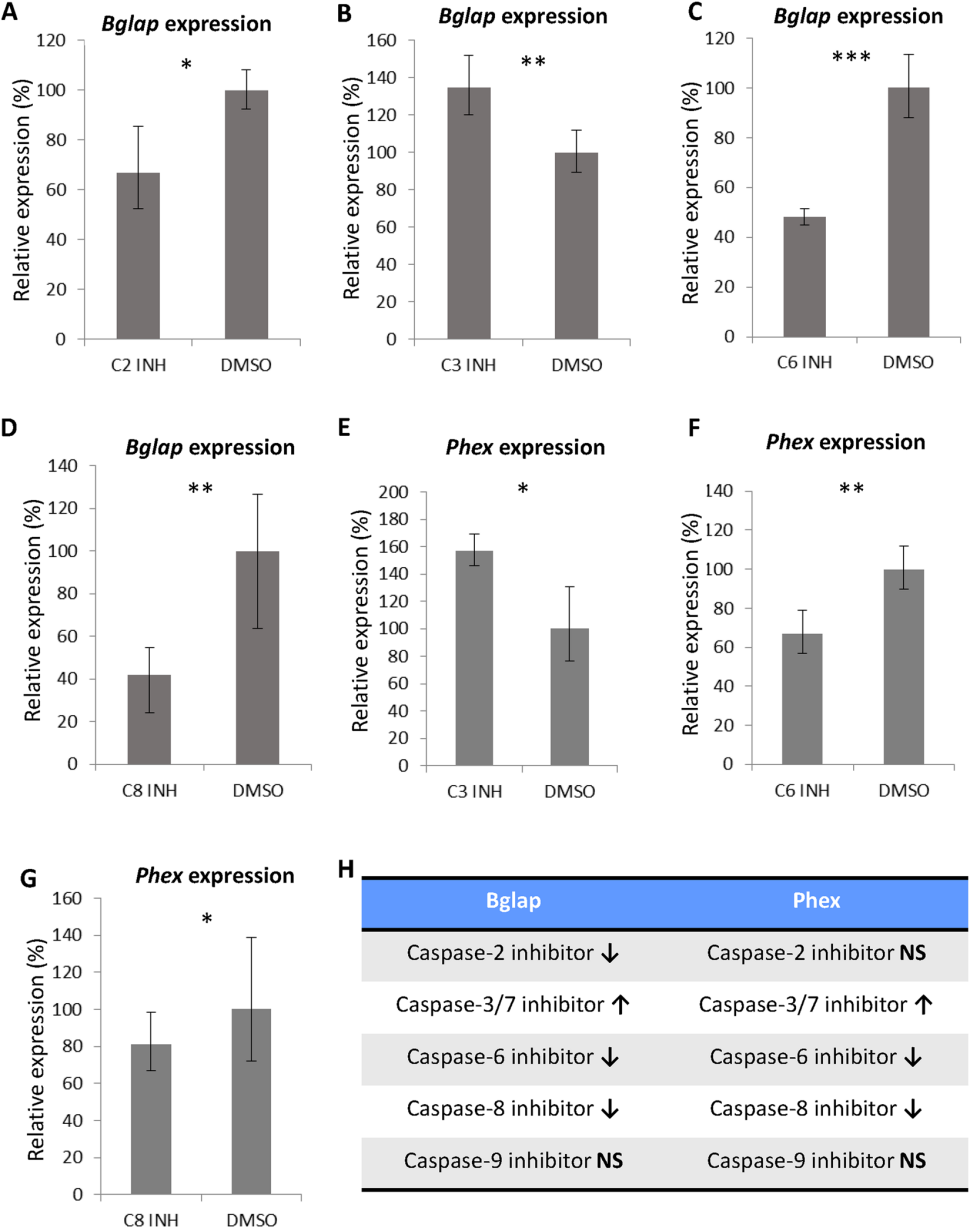
*Bglap* (**A–D**) and *Phex* (**E–G**) expression in the differentiated MC3T3-E1 cells after the inhibition of individual caspases. Expression levels were compared to expression in the control cells. Results are as a % indicating the mean±standard deviation of three replicates (expression in the control cells was set as 100%).* indicates *p* ≤ 0.05, ** indicates *p* ≤ 0.01, *** indicates *p* ≤ 0.001. Summary of inhibition of individual caspases (H), **↑** increasing expression after inhibition, **↓** decreasing expression after inhibition and **NS** non-significant.

In the inhibition experiments, differentiated MC3T3-E1 cells were first challenged by the general caspase inhibitor Z-VAD-FMK, and after 6 days, their osteogenic profile was compared to that of the control (DMSO treated). Of the panel of 84 genes, the expression of two genes, *Bglap* (the gene encoding osteocalcin) and *Phex* (phosphate-regulating neutral endopeptidase, X-linked gene), was significantly changed, more than 2-fold (Fig. 3A). The decrease in the regulation of these genes after caspase inhibition was confirmed by real-time PCR (P < 0.05) (Fig. 3B,C). To determine which apoptotic caspases were involved in *Bglap* and *Phex* regulation, individual caspase inhibitors were tested (Fig. 4). A statistically significant decrease in *Bglap* expression was observed after the inhibition of caspase-2 (P < 0.05), caspase-6 (P < 0.001) and caspase-8 (P < 0.01) (Fig. 4A,C,D). In contrast, the inhibition of caspase-3/7 caused a significant (P < 0.01) increase in *Bglap* expression (Fig. 4B). Similarly, the expression of *Phex* was also increased (P < 0.05) after the inhibition of caspase-3/7 (Fig. 4E). A decrease in the expression of *Phex* was found after the inhibition of caspase-6 (P < 0.01) and caspase-8 (P < 0.05) (Fig. 4F,G). The results of the individual caspase inhibition experiments are summarized in Fig. 4H.

Moreover, we observed an almost 2-fold decrease (1.87-fold, P < 0.05) in *Alpl* gene expression after general caspase inhibition (Fig. 3A). This decrease was slightly under the PCR Array threshold, which was based on a −/+2-fold change. Along with the downregulated expression of *Alpl* detected by PCR Arrays, alkaline phosphatase activity also decreased in the FMK inhibitor-treated group (Fig. 3D,E).

## Discussion

Pharmacological pan-caspase inhibition has recently been reported to significantly affect the expression of osteocalcin, a major marker of osteoblastic differentiation^4^. Furthermore, the possible engagement of proapoptotic caspases in cell differentiation has been reviewed^12^. Additionally, the nonapoptotic activation of caspases was demonstrated in MC3T3-E1 cells^4^, the most common *in vitro* models for osteoblastic lineage.

The differentiation of MC3T3-E1 cells is commonly achieved by the exposure of precursor cells to ascorbic acid^13,14^. Ascorbic acid-stimulated MC3T3-E1 cells synthesize and organize the collagenous matrix and undergo mineralization in manner very similar to that of bone *in vivo*^15^. Nevertheless, MC3T3-E1 cells cultured in AA/βGP-free medium can spontaneously undergo the differentiation process induced by cell-cell contact but are unable to completely differentiate^16^. The reason is attributed to collagen I transcription being independent of ascorbic acid induction; however, ascorbic acid is required for the deposition of collagen in the extracellular matrix^17^.

Recently, ascorbic acid was demonstrated to modulate signaling pathways in several cell types^18,19^. Therefore, to determine the impact of ascorbic acid induction on the expression of major osteogenic markers, the profile of the stimulated MC3T3-E1 cells was compared to that of the nondifferentiated MC3T3-E1 cells.

Differentiated MC3T3-E1 cells are characterized by high expression levels of alkaline phosphatase (Alp) and increasing activity during the early matrix production period^9,10^. As expected, the level of Alpl was found to be higher in the induced cells. In terms of other genes with changed osteogenic gene expression, the most prominently altered was *Tnfsf11*, the gene for RANKL. The increased production of RANKL was connected with more effective osteoblast differentiation^20^. Similarly, increased expression of the transcription factor Sp7 had an effect on osteoblast specification^21^, and Igf-1 was expressed in conjunction with MC3T3-E1 cell differentiation^13^. Additionally, in both groups of cultured cells, with and without AA/βGP, several genes were constantly expressed at high levels (Table 1). Thus, after ascorbic acid stimulation of the MC3T3-E1 cells, the changes in osteogenic markers were mostly quantitative. Therefore, the main difference in the two cell groups is not based on their rates or states of differentiation but on their mineralized matrix production, as previously reported^15^.

Since one of the most important osteoblastic markers, osteocalcin, was downregulated by general caspase inhibition^4^, the MC3T3-E1 cells were tested to obtain details on the impact of the individual caspases critical for this effect. Widely used specific FMK inhibitors^1^ were applied, and caspase-2,-3,-6,-7,-8, and-9 were investigated as major candidates with a broader spectrum of functions, in addition to apoptosis^2^.

The most important decrease in osteocalcin expression was caused by the inhibition of caspase-8. This caspase was previously determined as a molecule with a significant effect on MC3T3-E1 cell differentiation; however, osteocalcin was not evaluated in the previous study^5^. Furthermore, the inhibition of caspase-2, which can be a substrate for caspase-8 in nonapoptotic events^22^, also caused a decrease in osteocalcin expression. However, the inhibition of the intrinsic apical caspase, caspase-9, did not have any significant impact on osteocalcin expression in the MC3T3-E1 cells. These results were supported by the bioluminescence-based quantification of caspase activation during MC3T3-E1 cell differentiation. While the activation of caspase-9 ceased after the proliferation phase, the level of active caspase-8 increased, and this increased level was maintained until the final differentiation stage. An analysis of caspase activation during MC3T3-E1 cell differentiation was performed, for the first time, and the results indicated different roles for individual caspases during the progression of osteoblastic differentiation. These observations thus point to extrinsic components being crucial for the decreased osteocalcin levels after general caspase inhibition.

Among the executive caspases, caspase-6 inhibition displayed a negative effect on osteocalcin mRNA levels. The role of caspase-6 in differentiation has not yet been determined. However, caspase-6 is known to be involved in immune B-cell differentiation and to act in Tnf-alpha induction in macrophages^23,24^. Caspase-6 can cleave Satb1^25^, a protein from the same family as Satb2, which regulates osteoblastic differentiation^26^. Moreover, a lower level of Satb2 protein resulted in decreased levels of Runx2, Osx, Bsp and osteocalcin mRNA^27^. Recently, caspase-dependent cleavage of Satb2 was observed during the differentiation processes (preprinted work)^28^.

On the other hand, the inhibition of caspase-3/7 had an opposite effect on osteocalcin expression in the MC3T3-E1 cells, indicating specific roles for caspase-3 and caspase-7 connected with their major functions in apoptosis. Caspase-3 and caspase-7 are known to be connected with the intrinsic apoptotic pathway; however, along with caspase-9, they have distinct roles in this process^29^. Unfortunately, due to overlapping substrate specificity^30^, FMK inhibition of caspase-3 and caspase-7 cannot be performed separately.

Decreased expression of *Phex* was observed as was that of osteocalcin. Phex is a transmembrane molecule expressed by osteoblasts and its deletion is linked to hypophosphatemia^31,32^. In the individual caspase inhibition experiments, *Phex* expression was downregulated as a consequence of caspase-8 and caspase-6 inhibition, similar to osteocalcin.

In osteoblasts, Phex is downregulated by exposure to parathyroid hormone (PTH)^33^. In fact, PTH induces the differentiation of MC3T3-E1 cells by activating the Wnt/β-catenin pathway^34^. Caspases-8,-6 and-3 are known to directly lead to β-catenin proteolysis *in vitro*^35^. Caspases may thus link these pathways.

In conclusion, pharmacological caspase inhibition in osteoblastic MC3T3-E1 cells can decrease the expression of two clinically important osteogenic factors: osteocalcin and Phex. In both cases, the nonapoptotic effects of caspase-8 and caspase-6 were involved in this process. *In vivo*, deregulated levels of osteocalcin or Phex have a great influence on bone homeostasis and disorders. For example, mutations in the Phex gene are connected with rachitic changes in the bone growth plate, and this effect is mimicked by caspase inhibitors^36^. Osteocalcin is clinically important not only in bone regulation but also in many other tissues where it has a mediating role^37^. The regulation of Phex expression by caspase inhibition was described for the first time as was the specification of individual caspases in osteocalcin and Phex pathways. The apparent contribution of proapoptotic caspases and the possibility of their targeted inhibition can be considered in future therapeutic strategies.

## Materials and Methods

### Cell line and culture

The MC3T3-E1 cell line was purchased from Sigma-Aldrich (99072810) and cultivated in a nondifferentiation medium consisting of MEM alpha (A1049001, Gibco), 10% fetal bovine serum (F2442, Sigma-Aldrich) and penicillin/streptomycin (1000 U/ml, 100 μg/ml). The medium was replaced every 2–4 days, and the cells were passaged upon reaching 80% confluence.

For the experiments, passages 6–20 were used. The cells were seeded and cultured for 21 days in nondifferentiation and differentiation conditions in parallel. Differentiation medium was prepared as described above but with the addition of 10 mM β-glycerolphosphate (βGP) and 50 µg/ml ascorbic acid (AA). The cultured cells were passaged several times to avoid the formation of a dense collagenous matrix. After 3 weeks of culture, the cells were either fixed on plates with 4% PFA and stained with alizarin red, lysed in RLT buffer or used for caspase inhibition experiments, as described below.

### Caspase inhibition

After 21 days, differentiated cells were used for caspase inhibition. The cells were seeded at a density of 5000 cells per cm^2^ and cultured for 6 days (as reported previously^4^) in the presence of caspase inhibitors: general caspase inhibitor Z-VAD-FMK and inhibitors for individual apoptotic caspases (Caspase-2,-3,-6,-8,-9). Pharmacological inhibitors (FMK001, FMK003, FMK004, FMK006, FMK007, and FMK008, R&D Systems) were added to the culture medium at a concentration of 100 μM, according to the manufacturer’s instructions and previous studies^4^. Controls were generated using DMSO as an inhibitor vehicle at the same concentration. The medium with different treatments was changed every 2 days. The effectiveness of the individual caspase inhibitors was verified with Caspase-Glo assays (Supplement 2), and the constant inhibition effect of the FMK inhibitors in cells was confirmed previously^38^

### RNA isolation, real-time PCR, PCR Arrays

Cells for RNA isolation were lysed in 350 µl of RLT buffer with β-mercaptoethanol. RNA was isolated by using a RNeasy Mini Kit (Qiagen). First-strand cDNA was transcribed using Super Script VILO (Invitrogen); qPCR was performed in 10 μl of a final reaction mixture containing the one-step GB Ideal PCR Master Mix (Generi Biotech), Osteocalcin (Mouse *Bglap*, Mm03413826_mH), and* Phex* (Mouse Phex, Mm00448119_m1) expression was detected by using a TaqMan Gene Expression Assay (Thermo Fisher Scientific). The expression levels were calculated using the ∆∆CT method with normalization based on actin levels (Mouse Actb, Mm02619580_g1).

Osteogenic-related gene expression was analyzed by RT^2^ Profiler PCR Array Mouse Osteogenesis (PAMM026Z, Qiagen), which allows for expression comparisons of 84 genes.

### Caspase activity assay

Caspase activity was measured with Caspase-Glo bioluminescence assays (Promega) using a laboratory-built luminometer suitable for single-cell caspase detection, as described previously^39^. Currently, a Caspase-Glo reaction kit is available only for caspase-3/7,-8 and-9. The caspase-3/7,-8 and-9 activity levels were measured during the differentiation of MC3T3-E1 cells on day 1 and then periodically after every 7 days until the end of the differentiation phase on day 21. After being harvested by trypsinization, the cells were centrifuged and suspended in fresh medium. Under phase-contrast microscopic inspection, 50 cells were collected using an ICSI micromanipulator (Eppendorf) equipped with a 20-μm o.d. holding micropipette (Microtech) and transferred to 5-μL glass vials filled with 4 μL of Caspase-Glo; this reagent contained 60 μM MG-132 proteasome inhibitor (Promega) to suppress the background signal. The vials were incubated at room temperature, and the signal intensity was periodically measured until the bioluminescence emission reached a steady state. All measurements were performed in triplicate separately for each caspase.

### Staining for alkaline phosphatase

Fixed cells were stained with 300 μl of Fast blue mixture containing 4 mg of naphthol AS-TR phosphate disodium salt (Sigma, N6125) in 150 μl of N,N-dimethylformamide (Fluka, 40248) and 12 mg of Fast blue BB Salt hemi(zinc chloride) salt (Sigma, F3378) in 15 ml of 0.1 M Tris-HCl buffer (pH 9.6) for 10 min in the dark.

### Statistical analysis

Real-time PCR data were assessed using the ∆∆CT method. Three independent biological samples were analyzed, and the reactions were performed in triplicate for each sample. Expression level was related to that of the control cells, and the differences were evaluated using a t-test. The threshold for statistical significance was set to P < 0.05.

The expression data of the PCR Array were statistically evaluated by the Qiagen Data Analysis Center using a t-test, as recommended by the producer. Three independent biological samples were analyzed for each stage. The housekeeping genes included Actb, B2m, Gapdh, Gusb, and Hsp90ab1. Significance was determined as P < 0.05, and the threshold for fold changes was ±2.

Caspase activity levels were statistically analyzed in a Statistica software system (version 13, Dell Inc., 2016) using one-way ANOVAs, and Tukey HSD was used as a posthoc test. The threshold for significance was set to P < 0.05. Three independent samples were analyzed for each time point.

## Supplementary information

Supplementary files 1 and 2.

## References

[1] Kudelova J, Fleischmannova J, Adamova E, Matalova E (2015). Pharmacological caspase inhibitors: research towards therapeutic perspectives. J. Physiol. Pharmacol..

[2] Shalini S, Dorstyn L, Dawar S, Kumar S (2015). Old, new and emerging functions of caspases. Cell Death Differ..

[3] Miura M (2004). A crucial role of caspase-3 in osteogenic differentiation of bone marrow stromal stem cells. J. Clin. Invest..

[4] Svandova E, Vesela B, Tucker AS, Matalova E (2018). Activation of Pro-apoptotic Caspases in Non-apoptotic Cells During Odontogenesis and Related Osteogenesis. Front. Physiol..

[5] Mogi M, Togari A (2003). Activation of Caspases Is Required for Osteoblastic Differentiation. J. Biol. Chem..

[6] Ivaska KK (2005). Urinary osteocalcin as a marker of bone metabolism. Clin. Chem..

[7] Beck GR, Zerler B, Moran E (2001). Gene Array Analysis of Osteoblast Differentiation. Cell Growth Differ..

[8] Raouf A, Seth A (2002). Discovery of osteoblast-associated genes using cDNA microarrays. Bone.

[9] Choi J-Y (1996). Expression patterns of bone-related proteins during osteoblastic differentiation in MC3T3-E1 cells. J. Cell. Biochem..

[10] Sudo H (1983). *In vitro* differentiation and calcification in a new clonal osteogenic cell line derived from newborn mouse calvaria. J. Cell Biol..

[11] Czekanska E, Stoddart M, Richards R, Hayes J (2012). In search of an osteoblast cell model for *in vitro* research. Eur. Cells Mater..

[12] Pérez-Garijo A (2018). When dying is not the end: Apoptotic caspases as drivers of proliferation. Semin. Cell Dev. Biol..

[13] Thrailkill KM, Siddhanti SR, Fowlkes JL, Quarles LD (1995). Differentiation of MC3T3-E1 Osteoblasts is associated with temporal changes in the expression of IGF-I and IGFBPs. Bone.

[14] Yan X-Z (2014). Effects of Continuous Passaging on Mineralization of MC3T3-E1 Cells with Improved Osteogenic Culture Protocol. Tissue Eng. Part C Methods.

[15] Addison WN (2015). Extracellular matrix mineralization in murine MC3T3-E1 osteoblast cultures: An ultrastructural, compositional and comparative analysis with mouse bone. Bone.

[16] Miron RJ (2013). Influence of Enamel Matrix Derivative on Cells at Different Maturation Stages of Differentiation. PLoS One.

[17] Quarles LD, Yohay DA, Lever LW, Caton R, Wenstrup RJ (1992). Distinct proliferative and differentiated stages of murine MC3T3-E1 cells in culture: An *in vitro* model of osteoblast development. J. Bone Miner. Res..

[18] Wohlrab C (2018). The Association Between Ascorbate and the Hypoxia-Inducible Factors in Human Renal Cell Carcinoma Requires a Functional Von Hippel-Lindau Protein. Front. Oncol..

[19] Wohlrab C (2019). Ascorbate modulates the hypoxic pathway by increasing intracellular activity of the HIF hydroxylases in renal cell carcinoma cells. Hypoxia.

[20] Atkins GJ (2003). RANKL Expression Is Related to the Differentiation State of Human Osteoblasts. J. Bone Miner. Res..

[21] Hojo H, Ohba S, He X, Lai LP, McMahon AP (2016). Sp7/Osterix Is Restricted to Bone-Forming Vertebrates where It Acts as a Dlx Co-factor in Osteoblast Specification. Dev. Cell.

[22] Tsapras P, Nezis IP (2017). Caspase involvement in autophagy. Cell Death Differ..

[23] Watanabe C, Shu GL, Zheng TS, Flavell RA, Clark EA (2008). Caspase 6 regulates B cell activation and differentiation into plasma cells. J. Immunol..

[24] Ladha S (2018). Constitutive ablation of caspase-6 reduces the inflammatory response and behavioural changes caused by peripheral pro-inflammatory stimuli. Cell Death Discov..

[25] Galande S, Dickinson LA, Mian IS, Sikorska M, Kohwi-Shigematsu T (2001). SATB1 Cleavage by Caspase 6 Disrupts PDZ Domain-Mediated Dimerization, Causing Detachment from Chromatin Early in T-Cell Apoptosis. Mol. Cell. Biol..

[26] Dobreva G (2006). SATB2 Is a Multifunctional Determinant of Craniofacial Patterning and Osteoblast Differentiation. Cell.

[27] Zhang J (2011). Roles of SATB2 in Osteogenic Differentiation and Bone Regeneration. Tissue Eng. Part A.

[28] Bell, R. A. V. *et al*. Chromatin reorganization during myoblast differentiation involves the caspase-dependent removal of SATB2. *bioRxiv* 2019.12.19.883579 10.1101/2019.12.19.883579 (2019). 10.3390/cells11060966 PMC8946544 35326417

[29] Brentnall M, Rodriguez-Menocal L, De Guevara R, Cepero E, Boise LH (2013). Caspase-9, caspase-3 and caspase-7 have distinct roles during intrinsic apoptosis. BMC Cell Biol..

[30] Fuentes-Prior P, Salvesen GS (2004). The protein structures that shape caspase activity, specificity, activation and inhibition. Biochem. J..

[31] Ruchon AF (1998). Pex mRNA is localized in developing mouse osteoblasts and odontoblasts. J. Histochem. Cytochem..

[32] Yuan B (2008). Aberrant Phex function in osteoblasts and osteocytes alone underlies murine X-linked hypophosphatemia. J. Clin. Invest..

[33] Alos N, Ecarot B (2005). Downregulation of osteoblast Phex expression by PTH. Bone.

[34] Tian Y, Xu Y, Fu Q, He M (2011). Parathyroid hormone regulates osteoblast differentiation in a Wnt/β-catenin-dependent manner. Mol. Cell. Biochem..

[35] Van de Craen M (1999). Proteolytic cleavage of β-catenin by caspases: an *in vitro* analysis. FEBS Lett..

[36] Sabbagh Y, Carpenter TO, Demay MB (2005). Hypophosphatemia leads to rickets by impairing caspase-mediated apoptosis of hypertrophic chondrocytes. Proc. Natl. Acad. Sci..

[37] Diaz-Franco M, Franco-Diaz de Leon R, Villafan-Bernal J (2019). Osteocalcin-GPRC6A: An update of its clinical and biological multi-organic interactions (Review). Mol. Med. Rep..

[38] Chlastakova I (2012). Dynamics of caspase-3 activation and inhibition in embryonic micromasses evaluated by a photon-counting chemiluminescence approach. Vitr. Cell. Dev. Biol. - Anim..

[39] Ledvina V, Janečková E, Matalová E, Klepárník K (2017). Parallel single-cell analysis of active caspase-3/7 in apoptotic and non-apoptotic cells. Anal. Bioanal. Chem..

