# Supplementary material for: Osteogenic impact of pro-apoptotic caspase inhibitors in MC3T3-E1 cells
Source: Sci Rep. 2020 May 4;10:7489. doi: 10.1038/s41598-020-64294-9 (PMC7198622; doi:10.1038/s41598-020-64294-9)
Supplement: Supplementary file 1 — Supplementary files 1 and 2. [file 41598_2020_64294_MOESM1_ESM.docx]

**Osteogenic impact of pro-apoptotic caspase inhibitors in MC3T3-E1 cells**

Adéla Kratochvílová ^1,2^, Barbora Veselá ^1*^, Vojtěch Ledvina^2,3^, Eva Švandová ^1,^, Karel Klepárník ^3^, Kateřina Dadáková ^2^, Petr Beneš ^2,4^, Eva Matalová ^1,5^

^1^Institute of Animal Physiology and Genetics, Academy of Sciences, Brno, Czech Republic

^2^Faculty of Science, Masaryk University, Brno, Czech Republic

^3^Institute of Analytical Chemistry of the Czech Academy of Sciences, Brno, Czech Republic

^4^International Clinical Research Center, St. Anne's University Hospital, Brno, Czech Republic

^5^Department of Physiology, University of Veterinary and Pharmaceutical Sciences, Brno, Czech Republic

*Corresponding author:

Barbora Vesela

[veselab.lab@gmail.com](mailto:veselab.lab@gmail.com)

IAPG, CAS, v.v.i

Veveri 97,

Brno 60200

Czech Republic

keywords: caspase inhibition, MC3T3-E1, osteoblasts, osteocalcin, Phex

| Casp 3/7 | 0 | 7 | 14 |
| --- | --- | --- | --- |
| 7 | 0.966 |  |  |
| 14 | 0.973 | 1.000 |  |
| 21 | 0.028 | 0.015 | 0.016 |
| Casp 8 | 0 | 7 | 14 |
| 7 | 0.038 |  |  |
| 14 | 0.012 | 0.842 |  |
| 21 | 0.004 | 0.393 | 0.828 |
| Casp 9 | 0 | 7 | 14 |
| 7 | 0.992 |  |  |
| 14 | 0.020 | 0.029 |  |
| 21 | 0.299 | 0.419 | 0.272 |

**Supplement 1.** Significance of differences in caspase activities between monitored time points (days 0, 7, 14, and 21) showed as *p*-values. Significant differences are red.

**Supplement 2.** The efficacy of specific caspase inhibitors was verified using recombinant active caspases. The results of recombinant caspase-3 are shown in the graph as an example. The value 0.5 U of caspase-3 was used for the verification of an inhibitor efficacy. This amount was intentionally chosen to highly exceed the amount of the active enzyme within the measured sample of cells. Regarding the experiments, 0.5 µL (0.5 U) of the recombinant caspase-3 was mixed with 0.5 µL of the decimally diluted Z-DEVD-FMK caspase-3 inhibitor and 4 µL of the Caspase-Glo 3/7 reagent (Promega). The final concentrations of an inhibitor in the reaction mixture ranged from 100µM to 100pM. The results clearly demonstrate that 100-fold lower concentration of 1µM inhibitor is still sufficient enough to inhibit 0.5 U of purified caspase-3. Therefore, 100µM inhibitor should provide sufficient inhibition of the caspase-3 within the cell culture. This concentration is also recommended by the manufacturer.
